# Magnetostrictive Properties of the Grain-Oriented Silicon Steel Sheet under DC-Biased and Multisinusoidal Magnetizations

**DOI:** 10.3390/ma12132156

**Published:** 2019-07-04

**Authors:** Xiaojun Zhao, Yutong Du, Yang Liu, Zhenbin Du, Dongwei Yuan, Lanrong Liu

**Affiliations:** 1 Department of Electrical and Electronic Engineering, North China Electric Power University, Baoding 071003, China; 2 State Key Laboratory of Advanced Transmission Technology, Global Energy Interconnection Research Institute Co. Ltd., Beijing 102211, China; 3 Hebei Provincial Key Laboratory of Electromagnetic & Structural Performance of Power Transmission and Transformation Equipment, Baoding 071056, China

**Keywords:** GO silicon steel sheet, multisinusoidal magnetization, DC-bias, magnetostriction, acoustic noise level, three-limb laminated core, vibration

## Abstract

As an intrinsic property, elasticity of soft material is affected significantly by the externally applied alternating magnetic field. Magnetostrictive properties of the grain-oriented (GO) silicon steel under DC-biased and multisinusoidal magnetizations are measured by using a laser-based measuring system. Magnetostriction curves of the GO silicon steel sheet under different magnetizations are obtained and the influence of frequency and DC bias on the magnetostrictive property is observed and analyzed based on the measured data. In addition, the spectrum of magnetostriction under harmonic magnetization is obtained, and the acoustic noise level of the GO silicon steel sheet represented by the A-weighted decibel value caused by magnetostriction is measured under DC-biased and multisinusoidal magnetizations. The measurement results are applied to the simulation of the three-limb laminated core model, and the effects of DC bias and harmonics on magnetic flux density and displacement are analyzed.

## 1. Introduction

Magnetostrictive properties of electrical steel sheet under complicated excitations, such as harmonic excitation, DC-biased excitation, and even other extreme excitations, are of great importance for the analysis and design of large electromagnetic devices, especially for ultra-high voltage (UHV) power transformers and converters, and their performance highly affect the safe, economic, and reliable operation of a power system [1,2,3,4]. Owing to the demand for a high-capacity, long-distance, and high-voltage level power transmission, the ultra-high voltage direct current (UHVDC) transmission project has been in operation in China. Because of the particularity of the working conditions, a flood of harmonics flow into the windings of the smoothing reactor and converter transformer [5,6,7]. Moreover, the existence of DC bias phenomenon in the UHVDC usually causes significant saturation in transformer cores, and gives rise to more violent vibration and heavier noise due to the magnetostrictive properties of silicon sheet steel [8,9,10].

Magnetostriction arises from the movement and rotation of magnetic domain walls and varies nonlinearly with magnetic flux density in soft magnetic materials, such as GO silicon steel, amorphous soft magnetic alloys, and nanocrystalline soft magnetic alloys [11,12,13,14]. The GO silicon steel is mainly used for manufacturing the laminated core of large power transformers [15,16]. Vibration of the laminated core under some extreme excitations not only reduces the service life of the power transformer, but also threatens the safe operation of the power system, and the consequently generated tremendous noise should be reduced to prevent environmental pollution [17,18]. DC-biased magnetization or seriously distorted flux density will aggravate the magnetostriction of the GO silicon steel sheet and the vibration of the laminated core, which will eventually increase the acoustic noise level of the power transformer [19]. The requirements of the technical indicators in the manufacture of large electromagnetic devices can be met by reducing the influence of magnetostriction of the GO silicon steel sheet on transformer vibration and environmental noise. Therefore, it is of great significance to investigate the magnetostrictive properties of electrical steel sheets under DC-biased and harmonic magnetizations.

Some previous research work covers the measurements of magnetostriction, modeling of magnetostrictive curves, and rotational magnetostrictive properties [20,21,22]. In a previous study [23], a single sheet tester was built to measure the magnetoelastic properties of electrical sheet steel and simulated by using the two-dimensional finite element method. Anisotropic magnetostrictive properties of a highly GO electric steel sheet were measured by using a new round-type two-directional single sheet tester, and an improved method was proposed to model the magnetostrictive characteristics [24]. The magnetostriction of non-oriented electrical steel sheet under mechanical stress was measured by a six-axial strain gauge [25]. In another study [26], the influence of stresses and deformation on an electrical steel sheet was investigated and an experimental method for measuring the magnetoelastic properties was presented. The vibration of transformer cores caused by the magnetostriction of electrical steel sheets was investigated intensively [27]. The vibration noise effects of three-phase transformer were studied in another study [28], and the results implied that the harmonics in the load-current had a great effect on the vibration noise. The approach of reducing the noise and vibration were provided through the simulation of a transformer with a large capacity of 200 MVA (megavolt-ampere) [29]. In another study [30], the vibration of the super high-voltage generator transformer was analyzed and the complicated propagation path of the vibration in the tank and the transformer oil and the damping coefficient were all considered. DC bias experiments on AC power transformers were carried out, and the results showed that the waveform and spectrum of vibration and sound pressure of the power transformer were strongly affected by the DC bias [31].

Most of the current research focuses on the effect of alternating magnetization and mechanical stress on magnetostriction; however, DC bias and rich harmonics emerge due to the grid-connection of many UHVDC projects in China. Less attention has been paid to the effect of DC bias and harmonic magnetization on magnetostriction, and thus it is required to carry out an intensive investigation on modeling of the magneto-elastic properties of the GO silicon steel sheet and vibration characteristics of a laminated transformer core under non-sinusoidal magnetizations. In this paper, a laser-based measuring system is used to measure the magnetostrictive characteristics of the GO silicon steel sheets in the rolling direction under DC-biased and multi-harmonic magnetizations. The influence of DC bias and harmonic magnetic fields on magnetostrictive properties is analyzed based on the measured data obtained from experiments, which lays a foundation for investigating the mechanism of magnetostriction under DC-biased and harmonic magnetizations from the perspective of magnetic domain motion and rotation. Finite element computation is carried out to analyze the vibration characteristics of laminated sheets under sinusoidal, harmonic, and DC-biased magnetizations based on the coupled magneto-mechanical theory.

## 2. Materials and Methods

### 2.1. Measurement of One-Dimensional Magnetostrictive Properties

Several contact sensors were used to measure the magnetostriction of ferromagnetic materials. In order to overcome the disadvantage and limitations of the contact sensors, a contactless measuring system by means of a laser displacement sensor is developed and used in practical vibration measurement [32,33,34,35,36,37,38].

**Figure 1:**
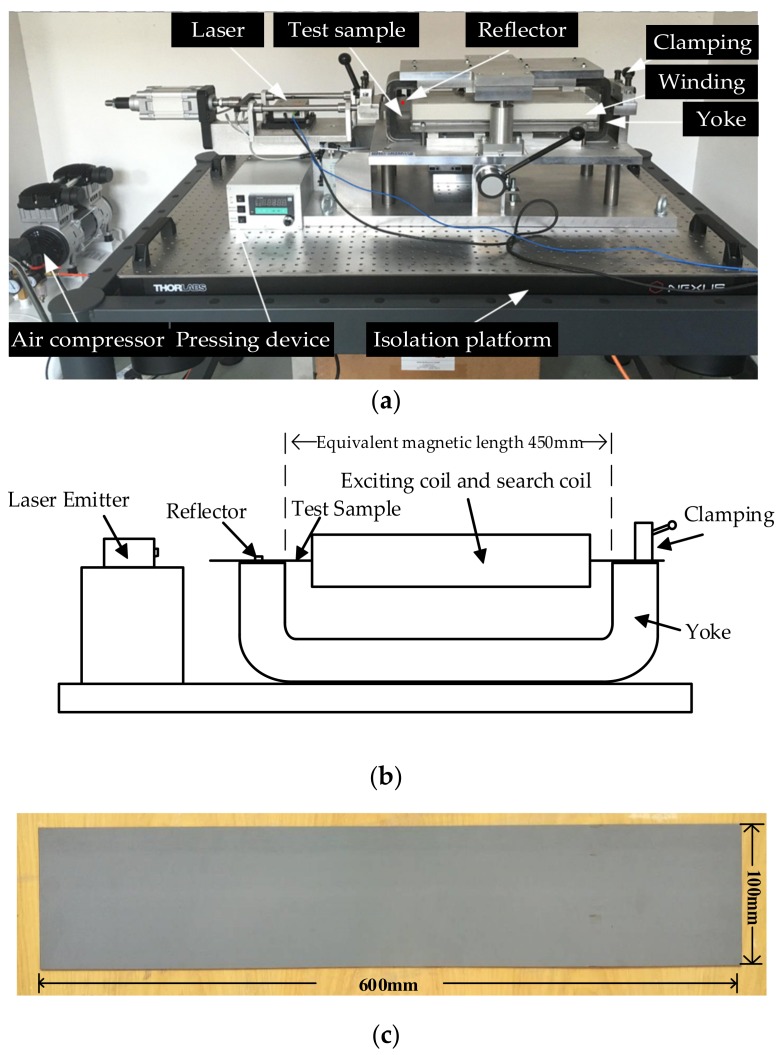
Laser-based measuring system of magnetostrictive properties: (**a**) Practical device; (**b**) diagrammatic sketch of measuring structure; (**c**) test sample.

The magnetostrictive properties of GO silicon steel sheets were measured by using a contactless measuring system demonstrated in Figure 1. This newly developed measuring system is produced by Brockhaus Group in Germany and the laser-based measurement is taken according to the Doppler principle. The measuring system, placed on a suspension platform supported by an air float, consists of the signal processing system, magnetic measuring structure, and laser transmitter. The magnetic measuring structure consists of two yokes, as well as the excitation coil (the number of turns is 370) and the search coil (the number of turns is 398). The test sample and lower yoke constitute the magnetic path, as demonstrated in Figure 1b. The sample of the GO silicon steel sheet (B30P105) with the dimensions of 600 × 100 × 0.3 mm is magnetized by the excitation coil. The equivalent magnetic length of this measuring system is defined as 450 mm. The length variation of the sample is determined by the detection of the distance between the reflector and transmitter after laser reflection. The laser-based measuring system can provide different types of excitations, such as sinusoidal, harmonic, and DC-biased excitations. The measuring system works in the range of 3 Hz to 1000 Hz. The demagnetization of the sample is considered in the magnetic measurement.

### 2.2. Noise Level Caused by Magnetostriction

Vibrations and the consequent noise of transformer cores arise from magnetostriction of the GO silicon steel sheet. Therefore, the acoustic characteristics of the GO silicon steel sheet should be observed to evaluate the influence of the magnetostriction on the noise. The A-weighted sound pressure level specified in IEC/TR 62581 [39], widely used in representation of audible sound, is adopted to evaluate the noise characteristics of the electrical steel sheet according to the following formulation:

(1) $$L_{VA}=20\log_{10}\frac{\rho_{0}\;c\sqrt{{\sum_{i}\left[{\left(2\pi \;f\right)}_{i}\cdot (\lambda_{i}/\sqrt{2})\cdot \alpha_{i}\right]}^{2}}}{p_{e0}}$$

where *L*_VA_ denotes the A-weighted decibel value; *ρ_0_* is the atmospheric density at room temperature; *c* is the sound velocity at room temperature; *f* is the fundamental frequency; *λ*_i_ is the amplitude of the *i*-th harmonic component of magnetostriction; *α*_i_ is the A-weighted coefficient corresponding to the *i*-th harmonic component; and *p*_e0_ is the minimum audible sound pressure.

### 2.3. Finite Element Equations of Coupled Magneto-Mechanical Theory for Laminated Steel Sheets

The phenomenon of magnetostriction occurs when the core is magnetized, the strain of the silicon steel sheet is related to the magnetic field, which results in the coupling between the magnetic and mechanical fields.

Based on Maxwell’s equations, the two-dimensional nonlinear magnetic field can be calculated by finite element and Galerkin methods as follows:

(2) $$\nabla \times \nu \nabla \times A+\sigma \frac{\partial A}{\partial t}=J$$

(3) $$SA+D\frac{\partial A}{\partial t}=G$$

(4) $$S_{pq}=\int_{\Omega }\nu \nabla N_{p}\cdot \nabla N_{q}d\Omega$$

(5) $$D_{pq}=\int_{\Omega }\sigma N_{p}N_{q}d\Omega$$

(6) $$G_{p}=\int_{\Omega }J\cdot N_{p}d\Omega$$

where ***S*** is the stiffness matrix related to magnetic field, ***A*** is the magnetic vector potential, ***D*** is the mass matrix related to magnetic field, ***J*** is the impressed current density, ***G*** is related to spatial distribution of the impressed current density, *σ* and *ν* are the conductivity and reluctivity, respectively. *p*, *q* = *i*, *j*, *m*. *N* is the shape function. *S_pq_*, *D_pq_* and *G_p_* are the representation of elements in ***S***, ***D***, and ***G***, respectively.

In the mechanical field, the fundamental equation of vibration, which ignores the damping term, can be expressed as follows:

(7) $$M\frac{\partial^{2}X}{\partial t^{2}}+KX=f_{\mathrm{em}}+f_{\mathrm{ms}}$$

where ***M*** is the mass matrix, ***K*** is the mechanical stiffness matrix, ***X*** is the displacement vector, ***f***_em_ is the Maxwell force, and ***f***_ms_ is the equivalent magnetostrictive force.

(8) $$M_{pq}=\rho h\iint_{\Omega }\left[\begin{array}{cc} N_{p}N_{q} & 0\\ 0 & N_{p}N_{q} \end{array}\right]d\Omega$$

(9) $$K_{pq}=\frac{E\left(1-\mu \right)h}{\left(1+\mu \right)\left(1-2\mu \right)}\iint_{\Omega }\left[\begin{array}{cc} \frac{\partial N_{p}}{\partial x}\frac{\partial N_{q}}{\partial x} & \frac{\mu }{1-\mu }\frac{\partial N_{p}}{\partial x}\frac{\partial N_{q}}{\partial y}\\ \frac{\mu }{1-\mu }\frac{\partial N_{p}}{\partial y}\frac{\partial N_{q}}{\partial x} & \frac{\partial N_{p}}{\partial y}\frac{\partial N_{q}}{\partial y} \end{array}\right]d\Omega$$

where *ρ* is density of silicon steel sheet, 7650 kg/m^3^; *h* is thickness of the three-limb laminated core model (provided in 3.3), 0.06 m. *E* is the Young’s modulus, 2 × 10^11^ Pa. *μ* is the Poisson ratio, 0.28. *M_pq_* and *K_pq_* are the representation of elements in matrix ***M*** and ***K***, respectively.

***f***_em_ is obtained by surface integral of the Maxwell stress tensor ***T***, and ***f***_ms_ of each element can be calculated by the stiffness matrix and displacement the using finite element method [12].

(10) $$f_{\mathrm{em}}=\;\oint_{s}T\cdot ds=-h\int_{\Omega }\left[\begin{array}{cc} B_{x}H_{x}-\frac{1}{2}BH & B_{x}H_{y}\\ B_{y}H_{x} & B_{y}H_{y}-\frac{1}{2}BH \end{array}\right]\left[\begin{array}{c} \frac{\partial N}{\partial x}\\ \frac{\partial N}{\partial y} \end{array}\right]d\Omega$$

(11) $$f_{\mathrm{ms}}{}^{e}=K^{e}\cdot x^{e}$$

where *B* is magnetic flux density, *H* is the magnetic field intensity. The subscript *x* and *y* denote the direction of coordinate axes.

The flux density of the element is considered to be distributed in the center of the element, the displacement ***x***^e^ due to magnetostriction is equal to the product of the distance *l* from the center of the element to the node and the magnetostrictive strain *ε*. The magnetostrictive strain is obtained by interpolation of the single-valued magnetostriction curve *λ*_pp_(*B*_m_), which is under sinusoidal excitation, provided in 3.3.

(12) $$x_{p}{}^{e}=\left(\begin{array}{c} l_{p,x}{}^{e}\cdot \varepsilon_{r(t)}{}^{e}\\ l_{p,y}{}^{e}\cdot \varepsilon_{r(t)}{}^{e} \end{array}\right)=\left(\begin{array}{c} l_{p,x}{}^{e}\cdot \lambda_{r(t)}{}^{e}\\ l_{p,y}{}^{e}\cdot \lambda_{r(t)}{}^{e} \end{array}\right)$$

where the subscript *r* refers to the rolling direction and *t* refers to the transverse direction, the subscript *x* and *y* refer to the direction of the coordinate axes.

## 3. Results and Discussion

### 3.1. Analysis of the Measured Magnetostrictive Properties

#### 3.1.1. Magnetostrictive Characteristics under DC-Biased Magnetization

In the experiment, the AC and DC excitations are applied simultaneously to make the GO silicon steel sheet magnetized under the DC bias condition. The alternating flux density *B_ac_* can be obtained from the induced voltage according to Faraday’s law:

(13) $$B_{ac}=\;\;\frac{1}{N_{\mathrm{coil}1}S}\int_{t_{1}}^{t_{2}}e\;\mathrm{dt}$$

where *S* is the cross-section area of one layer of the GO silicon steel sheet, *N*_coil1_ is the number of turns of the search coil, and *e* is the induced voltage.

The magnetic flux density can be expressed as follows:

(14) $$\begin{array}{l} B=B_{0}+B_{ac}\\ \;\;\;\;\;\;\;\;\;=B_{0}+B_{1}\sin\omega t \end{array}$$

where *B*_0_ is the DC flux density generated by the applied DC current excitation, *B*_1_ is the amplitude of the fundamental component of flux density generated by the applied alternating voltage excitation, and *ω* is the fundamental angular frequency. The fundamental frequency is 50 Hz in the experiment.

The magnetic field intensity *H* is obtained from the measured excitation current according to Ampere’s law, shown as follows:

(15) $$H=\;\;\frac{N_{\mathrm{coil}2}\cdot i}{L}$$

where *N*_coil2_ is the number of turns of the excitation coil, *L* is the equivalent magnetic length, which is 450 mm in measurement, and *i* is the excitation current.

It is noted that the DC flux and the corresponding DC component of flux density *B*_0_ in the silicon steel sheet cannot be obtained from Equation (13) directly, since it is really difficult to separate DC flux from AC-DC hybrid flux through measurement. However, quantization of the DC-biased magnetization can be realized by using the DC-biased magnetic intensity *H*_dc_, which can be computed according to Equation (15), since the applied DC current in the excitation coil is usually known in advance and can be measured easily.

**Figure 2:**
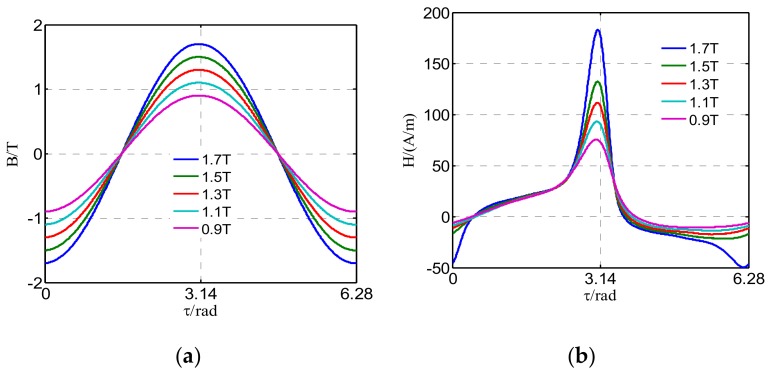

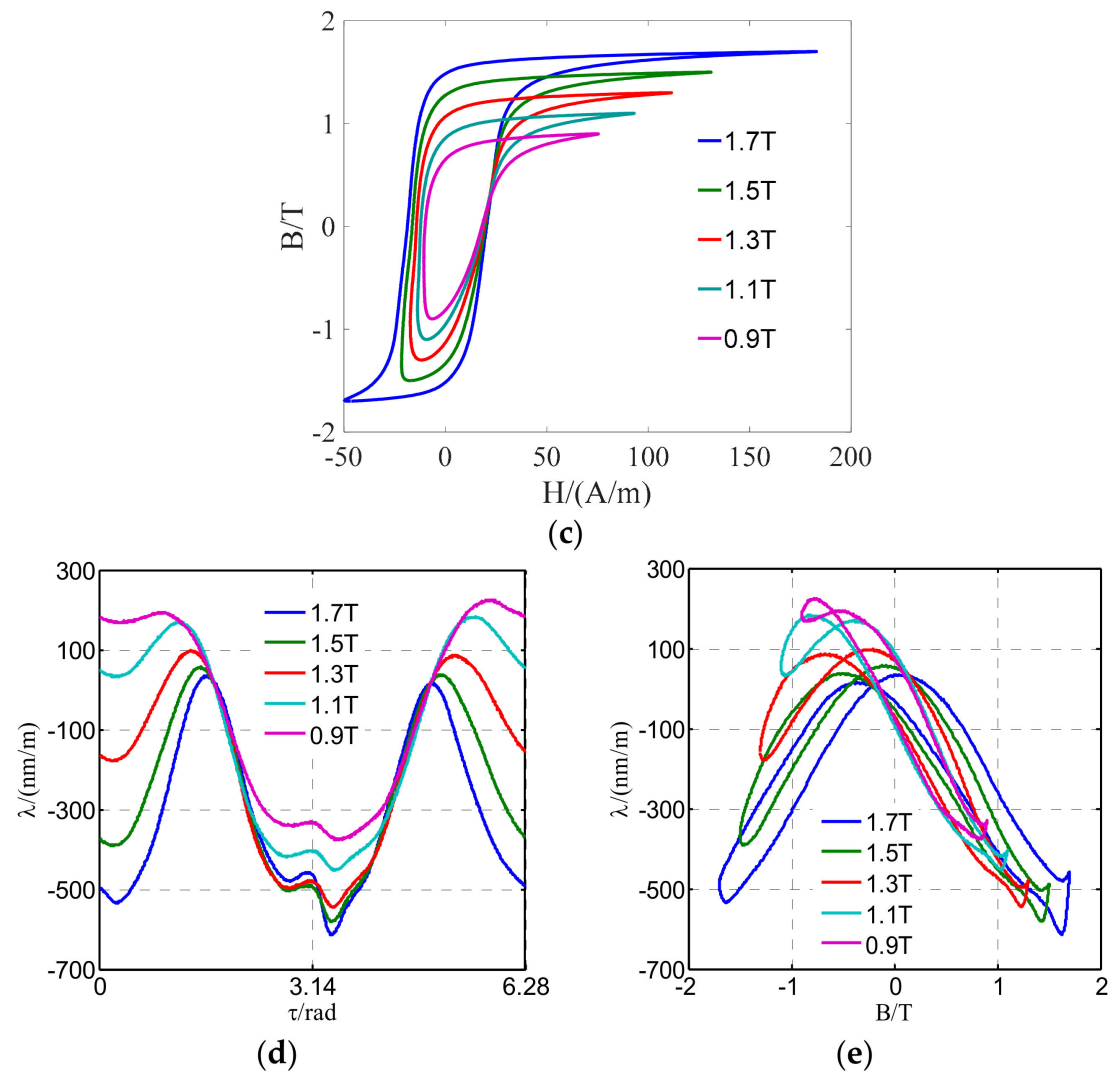
Measured results under DC-biased magnetization along the rolling direction (*H*_dc_ = 12 A/m, *B*_m_ = 0.9 T, 1.1 T, 1.3 T, 1.5 T, 1.7 T): (**a**) Waveforms of magnetic flux density; (**b**) waveforms of the measured magnetic intensity; (**c**) magnetic hysteresis loops; (**d**) waveforms of the measured magnetostrictive strain; (**e**) measured magnetostriction loops (butterfly curves).

Figure 2 shows the measured magnetic field and magnetostrictive strain when the DC-biased magnetic intensity *H*_dc_ is set to 12 A/m and the peak value of magnetic flux density *B*_m_ is controlled to be 0.9, 1.1, 1.3, 1.5, and 1.7 T.

The waveforms of magnetic flux density *B* and magnetic intensity *H* in the GO silicon steel sheet are shown in Figure 2a,b respectively. The waveforms of magnetic intensity are distorted seriously due to the significant nonlinearity of the GO silicon steel sheet. From Figure 2c, it can be seen that the magnetic hysteresis loops are not symmetric any more under DC bias.

Figure 2d depicts the measured magnetostrictive strain in one period. It can be seen that waveforms of magnetostriction of the silicon steel sheet are distorted significantly due to the DC-biased magnetization. The peak value of magnetostrictive strain varies with that of magnetic flux density along the rolling direction. Figure 2e shows that the magnetostrictive butterfly curve under DC-biased magnetization is not symmetric any more, and the “left wing” of the butterfly curve has a tendency of degenerating when the amplitude of flux density decreases [15]. This indicates that the influence of DC bias on the symmetry of the butterfly curve is significant, especially at low magnetic flux density.

**Figure 3:**
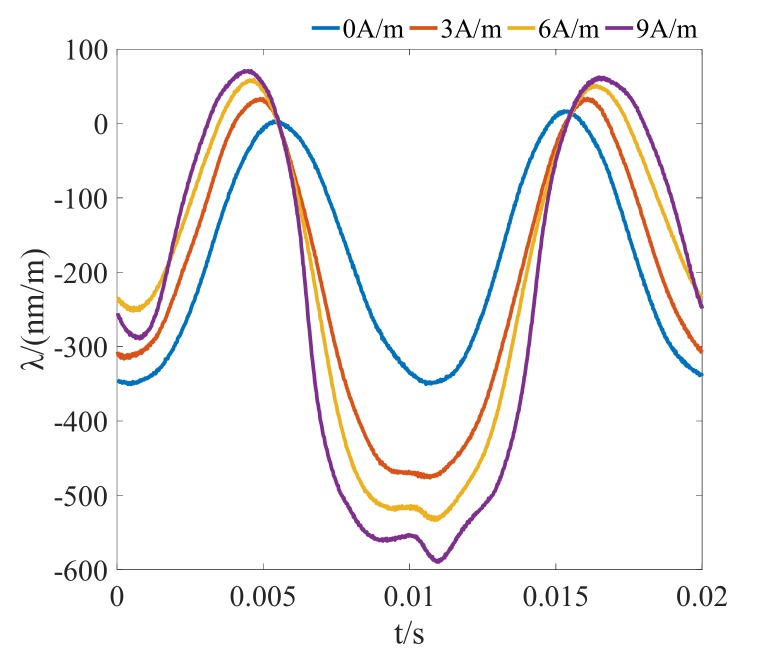
Magnetostriction versus time under different DC-biased magnetizations (*H*_dc_ = 0 A/m, 3 A/m, 6 A/m, 9 A/m, *B*_m_ = 1.3 T).

In addition, the DC-biased excitation has an obvious impact on the magnetostrictive strain. It can be seen from Figure 3 that the frequency of magnetostriction is 100 Hz under the sinusoidal excitation because the change rate of the length caused by magnetostriction mainly depends on the amplitude of magnetic flux density and relative direction between crystallographic axes of steel. However, more harmonics with higher frequencies are generated in the spectrum of the magnetostriction when the GO silicon steel sheet is magnetized by different DC-biased excitations. Moreover, it can be concluded that the presence of the DC-biased magnetic field increases the peak value of the magnetostrictive strain.

From the overall magnetostrictive property of the GO silicon steel sheet given in Figure 2 and Figure 3, it can be concluded that magnetostriction of the electrical steel sheet is affected by the DC-biased magnetization in two main aspects. Firstly, both the positive peak value (*λ*_p+_) and the negative peak value (*λ*_p−_) of the magnetostriction are affected by the alternating and DC-biased magnetic fields simultaneously. Furthermore, the difference between *λ*_p+_ and *λ*_p−_ becomes larger with the increasing DC bias. Secondly, magnetostriction exhibits special features of contraction and elongation under DC-biased magnetization. With the increase of the amplitude of flux density *B*_m_, the state of the silicon steel sheet corresponding to the negative peak value of the flux density gradually changes from elongation to contraction due to the applied DC-biased magnetic field.

**Figure 4:**
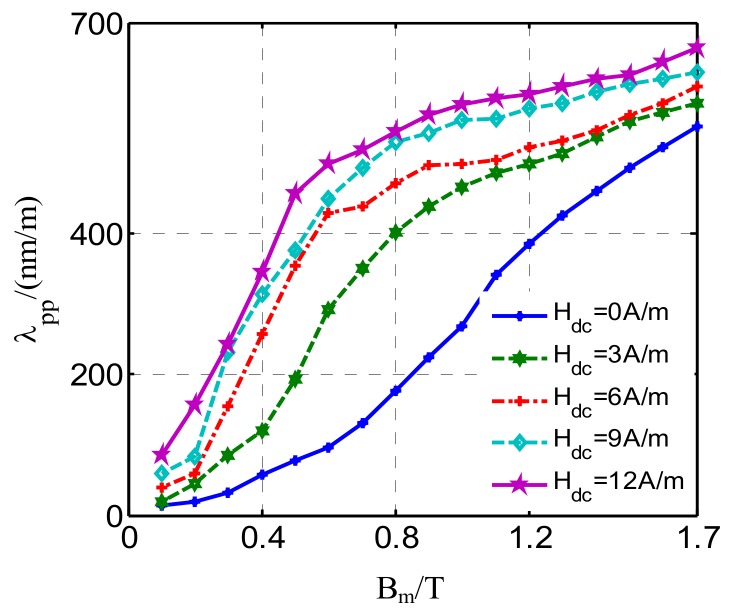
Magnetostriction curve under different DC-biased magnetizations (*H*_dc_ = 0 A/m, 3 A/m, 6 A/m, 9 A/m, 12 A/m).

The peak-to-peak value of magnetostriction *λ*_pp_ can be obtained from the difference between *λ*_p+_ and *λ*_p−_, thus a series of single-valued magnetostriction curves *λ*_pp_(*B*_m_) under different DC-biased magnetizations can be depicted, as shown in Figure 4. With the increase of DC bias, the magnetostriction curve moves upward. It can be concluded that the magnetostriction also has saturated characteristics due to the reduction in the number of active domain walls with the saturating tendency of the magnetic field. This reveals why large DC bias leads to violent vibration and even power transformer damage in UHVDC transmission.

#### 3.1.2. Magnetostrictive Characteristics under Harmonic Magnetization

The relationship between harmonic magnetization and magnetostriction of the GO steel sheet is investigated by applying a harmonic voltage excitation to magnetize the specimen in the measuring system. In this paper, only one high-order harmonic is superposed to the fundamental component due to the capacity limitation of the power supply. According to Faraday’s law shown in Equation (13), the harmonic voltage and the consequently generated harmonic flux density can be expressed as follows:

(16) $$U=U_{1}\cos(\omega t)+U_{n}\cos(n\omega t)$$

(17) $$\begin{array}{l} B\;\;=B_{1}\sin(\omega t)+B_{n}\sin(n\omega t)\\ \;\;\;\;\;\;\;=B_{1}\sin(\omega t)+k_{n}B_{1}\sin(n\omega t) \end{array}$$

where *U*_n_ is the amplitude of *n*-th harmonic voltage; *n* is the harmonic number; *B*_1_ is the amplitude of fundamental component of flux density; *B_n_* is the amplitude of n-th harmonic flux density; and *k_n_* is the amplitude ratio of the *n*-th harmonic to the fundamental component.

**Figure 5:**
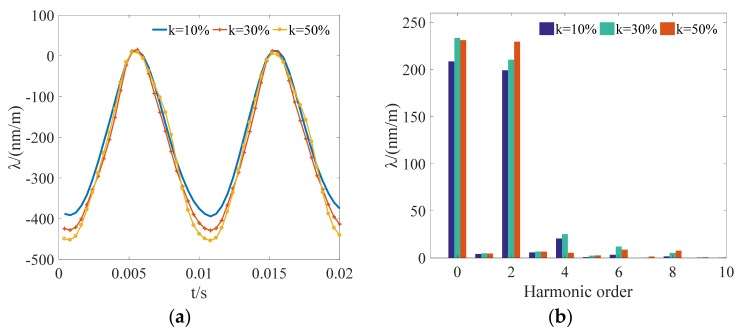
Measured results when the fifth harmonic is superposed to the fundamental component (*B*_m_ = 1.4 T, *k_5_* = 10%, 30%, 50%): (**a**) Waveforms of magnetostriction; (**b**) frequency spectrum of magnetostriction.

Figure 5a shows the measured magnetostrictive strain when the peak value of the flux density is kept at 1.4 T and the amplitude ratio is controlled at 10%, 30%, and 50%, respectively. The magnetostriction varies periodically at 100 Hz, and the peak value of magnetostriction increases with the content of the fifth harmonic. The 180° domain wall (DW) motion has little effect on magnetostriction, whereas the non-180° DW rotation gives rise to much larger magnetostricition. The measured magnetostriction of the GO silicon steel is very small, since the 180° DW movement predominates when the GO silicon steel is magnetized along the rolling direction (RD). Moreover, it can be inferred that non-180° DW rotation may play an important role in magnetostriction when the GO silicon steel is magnetized along the transverse direction (TD), which deserves more attention in analyzing the vibration characteristic of the transformer core under different excitations. Figure 5b shows that the corresponding spectrum of magnetostriction is mainly composed of a DC component and a second harmonic. Meanwhile, the fourth and other high-order harmonics account for a relatively small proportion.

**Figure 6:**
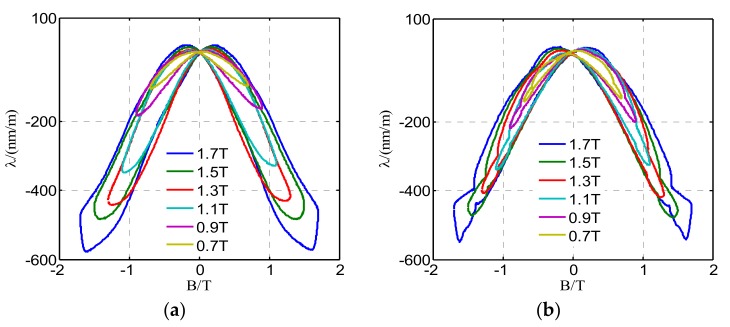
Measured butterfly curves under different harmonic magnetizations when the peak value of flux density *B_m_* varies from 0.7 T to 1.7 T: (**a**) Harmonic excitation, in which the third harmonic *B*_3_ accounts for 50% of the fundamental component *B*_1_; (**b**) harmonic excitation, in which the ninth harmonic *B*_9_ account for 50% of the fundamental component *B*_1_.

Figure 6 shows the measured butterfly curves under two different harmonic magnetizations, in which the third or the ninth harmonic accounts for 50% of the fundamental component. As can be seen from Figure 6, the curves remain symmetric, and the curves will be distorted seriously when a high-order harmonic is superposed to the fundamental component.

### 3.2. Noise under DC-Biased and Multisinusoidal Magnetizations

**Figure 7:**
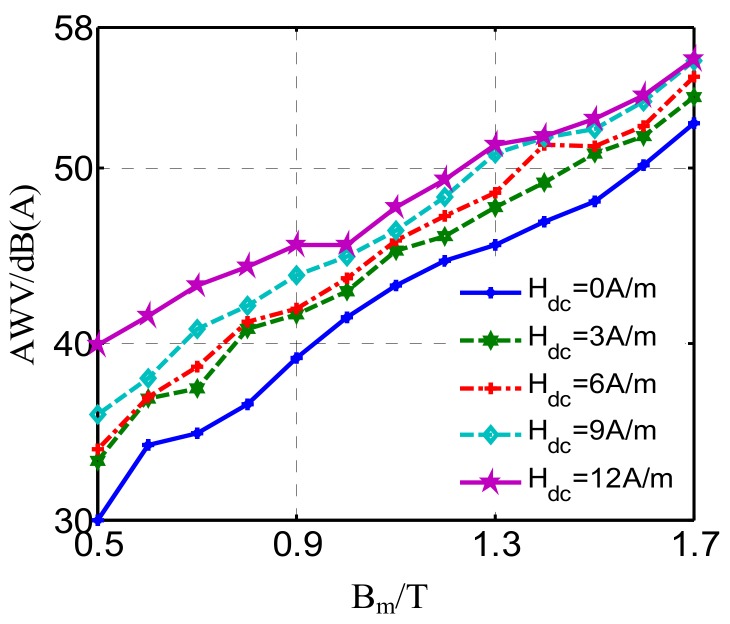
A-weighted noise value under different DC-biased magnetizations.

It is known that noise generated from the power transformer will increase significantly under DC bias conditions. Figure 7 shows the A-weighted noise level calculated by Equation (1) when applying different DC-biased excitations to the GO silicon steel sheet. The A-weighted noise value increases by 10 dB when the DC-biased magnetic field *H*_dc_ is enlarged from 0 A/m to 12 A/m under the condition of *B*_m_ = 0.5 T. Furthermore, the noise level under DC bias conditions is also dependent on *B*_m_, which indicates that the saturation of the transformer core due to DC bias will lead to larger environmental noise.

**Figure 8:**
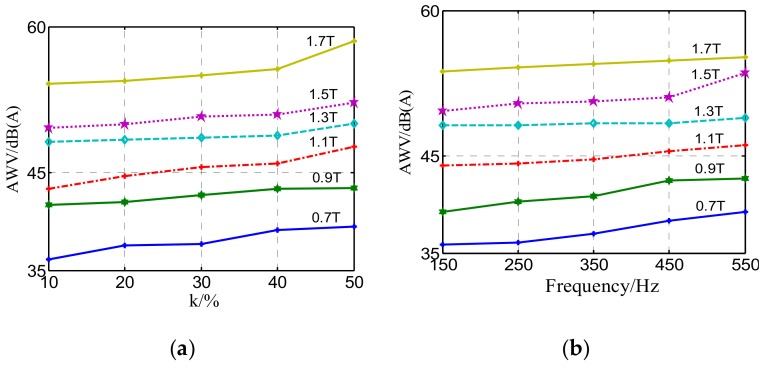
A-weighted noise value under different harmonic magnetizations: (**a**) Effect of varying harmonic content on noise (*n* = 5); (**b**) effect of superposed harmonic excitation frequency on noise when *k* = 30%.

A series of experiments and measurements were carried out to investigate the influence of harmonic content and frequency on the noise level of a GO silicon steel sheet. Figure 8a shows the increased harmonic content gives rise to larger environmental noise. The A-weighted noise value corresponding to *B*_m_ = 1.7 T is about 1.2 times of that corresponding to *B*_m_ = 1.1 T when the fifth harmonic accounts for 30% of the fundamental component in the hybrid harmonic excitation, and it is implied that the sound pressure increased by 2.8 times with *B*_m_ enlarged from 1.1 T to 1.7 T. As can be seen from Figure 8b, the higher the frequency is, the higher the noise will be when the amplitude ratio remains at 30%.

### 3.3. Simulation of Vibration Characteristic

**Figure 9:**
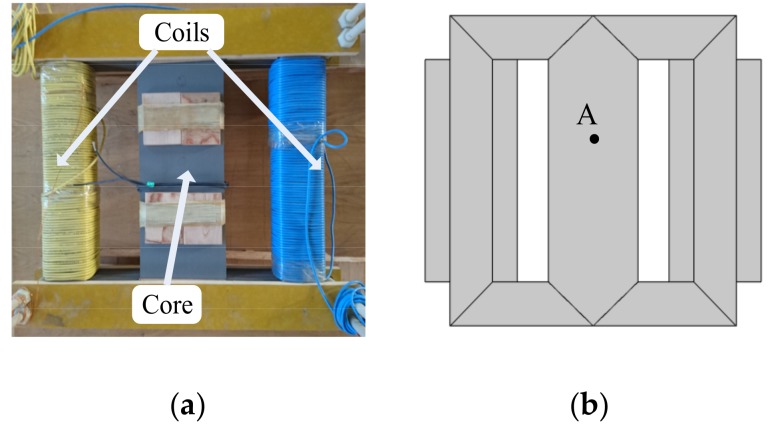
Three-limb laminated core: (**a**) Practical model; (**b**) simulated model.

**Figure 10:**
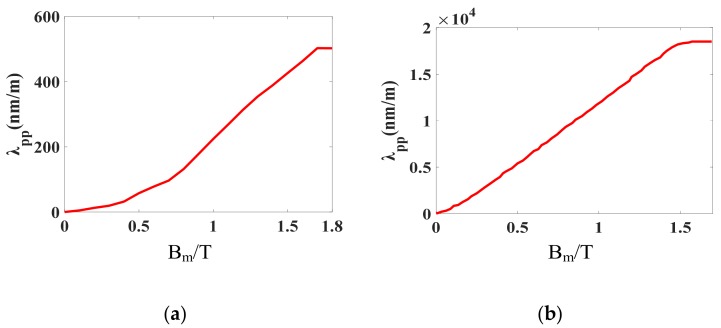
Single-valued magnetostriction curves: (**a**) Rolling direction; (**b**) transverse direction.

The three-limb laminated core made of silicon steel sheets (B30P105), which were previously used to measure magnetostriction, is investigated for vibration characteristics under sinusoidal, harmonic, and DC-biased magnetizations. The three-limb laminated core is shown in Figure 9a. The length, width, and thickness of the core are 600 mm, 575 mm, and 60 mm, respectively. There are two excitation coils in parallel on the side yoke, and the number of turns of each coil is 115. The two-dimensional model is structured and the coupled magneto-mechanical field is simulated. The single-valued magnetostriction curves *λ*_pp_(*B*_m_) along RD and TD under sinusoidal excitation are shown in Figure 10.

**Figure 11:**
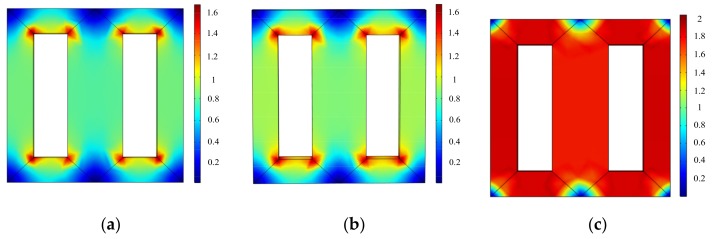
Magnetic flux density distributions of the laminated core: (**a**) Sinusoidal excitation (*B*_1_ = 0.8 T); (**b**) the fifth harmonic accounts for 50% of the fundamental component in the hybrid harmonic excitation (*B*_1_ = 0.8 T, *B*_5_ = 0.4 T); (**c**) DC bias of 1A (*B*_1_ = 0.8 T, *H*_dc_ = 76 A/m).

The magnetic flux density distributions of the laminated core at the time when flux density reaches its peak value in a period under sinusoidal, harmonic, and DC-biased magnetizations are shown in Figure 11. It can be seen that the magnetic flux density under harmonic and DC-biased conditions are larger than that under sinusoidal conditions; when the laminated core is magnetized under DC bias, the magnetic flux density shifts due to the DC component, resulting in larger magnetic flux density.

**Figure 12:**
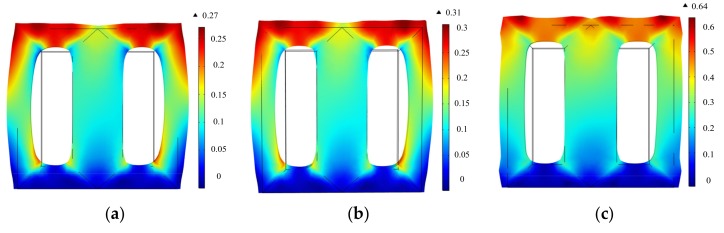
Displacement distributions of the laminated core: (**a**) Sinusoidal excitation (*B*_1_ = 0.8 T); (**b**) the fifth harmonic accounts for 50% of the fundamental component in the hybrid harmonic excitation (*B*_1_ = 0.8 T, *B*_5_ = 0.4 T); (**c**) DC bias of 1A (*B*_1_ = 0.8 T, *H*_dc_ = 76 A/m).

The bottom surface of the laminated core is fixed in the normal direction. The displacement distributions of the laminated core at the time when displacement reaches its peak value in a period under corresponding excitations are shown in Figure 12. The peak values of displacement of the core under sinusoidal, harmonic, and DC-biased excitations are 0.27 μm, 0.31 μm, and 0.64 μm, respectively. It can be seen that the harmonic and DC-biased phenomenon aggravate the vibration of the laminated core.

Table 1 shows the peak value of magnetic flux density and displacement on point A in a period under different magnetizations. It can be seen that the peak value of displacement increases with the content of the fifth harmonic and DC current. In addition, the difference between displacement with DC of 1.5A (*H*_dc_ = 113 A/m) and that with DC of 1A (*H*_dc_ = 76 A/m) is not obvious because the magnetostriction tends to be saturated when *B*_m_ is larger than 1.7 T.

**Figure 13:**
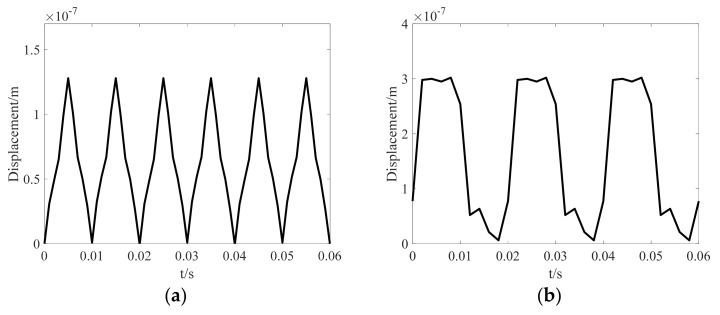
Waveforms of displacement on point A under different magnetizations: (**a**) The fifth harmonic accounts for 50% of the fundamental component (*B*_1_ = 0.8 T, *B*_5_ = 0.4 T); (**b**) DC bias of 1A (*B*_1_ = 0.8 T, *H*_dc_ = 76 A/m).

Figure 13 shows the waveforms of displacement on point A under harmonic and DC-biased magnetizations. The displacement varies periodically at 100 Hz when the fifth harmonic accounts for 50% of the fundamental component, and the period is 0.01 s. However, the displacement does not change twice at the DC bias of 1A (*H*_dc_ = 76 A/m) in a period of voltage, which varies periodically at 50 Hz.

## 4. Conclusions

Experiments are designed to carry out the measurement of magnetostrictive properties of the GO silicon steel sheet under DC-biased and harmonic magnetizations in this paper. The magnetostriction loops measured along the rolling direction are no longer symmetric under DC-biased magnetization; moreover, the DC-biased or harmonic magnetic fields gives rise to the increase of the peak value of magnetostriction. The peak-to-peak value of magnetostriction increases by 1.2 times with the DC-biased magnetic field *H*_dc_ of 12 A/m, and 1.1 times with the third harmonic accounting for 50% of the fundamental component in the hybrid harmonic excitation, respectively, compared with that under sinusoidal excitation when *B*_m_ = 1.7 T. In addition, the spectrum of magnetostriction is mainly composed of the DC component, second harmonic, as well as other high-order harmonics under DC bias or harmonic excitations, especially when the DC-biased excitation is applied to magnetize the electrical steel sheet. It can be concluded that the transformer core will vibrate within a wide range of frequencies.

The reduction of transformer noise largely depends on the improvement of the silicon steel sheet, and investigating the magnetostrictive and noise properties is beneficial to the acquisition of new de-noising methods. From the measurement of the acoustic noise level of the GO silicon steel sheet, it can be concluded that DC bias phenomenon and harmonic excitation will lead to more intense vibration in transformer cores and consequently generate very loud noise. Compared with the A-weighted noise value under sinusoidal excitation with *B*_m_ = 1.7 T, the measured results increases by 3.3 dB with the DC-biased magnetic field *H*_dc_ of 12 A/m, and 4.8 dB with the fifth harmonic, accounting for 50% of the fundamental component in harmonic excitation, respectively.

The simulated results show that DC-biased and harmonic phenomena aggravate the vibration of the three-limb laminated core. The higher the DC bias and the content of the harmonic, the heavier the vibration will be. The displacement increases by 2.78 times with the DC-bias of 1.5A (*H*_dc_ = 113 A/m), and 1.17 times with the fifth harmonic accounting for 50% of the fundamental component in the hybrid harmonic excitation, respectively, compared with that under sinusoidal excitation (*B*_1_ = 0.8 T). Therefore, it is required to attach more importance to the vibration and noise pollution of power transformers caused by DC bias phenomenon and harmonic excitation in HVDC.

## Figures and Tables

**Table 1:** Peak values of magnetic flux density and displacement on point A in a period under different magnetizations (*B*_1_ = 0.8 T).

	Peak Value of Magnetic Flux Density	Peak Value of Displacement
sinusoidal	0.777 T	1.0953 × 10^−7^ m
k_5_ = 10% (*B*_5_ = 0.08 T)	0.802 T	1.1479 × 10^−7^ m
k_5_ = 50% (*B*_5_ = 0.4 T)	0.852 T	1.2818 × 10^−7^ m
*I*_dc_ = 1A (*H*_dc_ = 76 A/m)	1.748 T	3.0191 × 10^−7^ m
*I*_dc_ = 1.5A (*H*_dc_ = 113 A/m)	1.793 T	3.0430 × 10^−7^ m

## References

[1] Price P.R. (2002). Geomagnetically induced current effect on transformers. IEEE Trans. Power Deliv..

[2] Pfutzner H., Shilyashki G., Bengtsson C., Trenner G., Gerstbauer E. (2018). Effects of dc bias on regional flux and magnetostriction of a single-phase transformer core modeled by 3-d macc. IEEE Trans. Magn..

[3] Baguley C.A., Madawala U.K., Carsten B. (2011). The impact of vibration due to magnetostriction on the core losses of ferrite toroidals under dc bias. IEEE Trans. Magn..

[4] Lu Z.M., Zhang C., Wang T.Z. Measurement and Analysis of UHV Transformer Noise with Sound Intensity and Vibration Method. Proceedings of the 20th International Conference on Electrical Machines and Systems (ICEMS).

[5] Mohammed O.A., Abed N.Y., Liu S. (2006). Investigation of the harmonic behavior of three phase transformer under nonsinusoidal operation using finite element and wavelet packets. IEEE Trans. Magn..

[6] Smajic J., Hughes J., Steinmetz T. (2012). Numerical computation of ohmic and eddy-current winding losses of converter transformers including higher harmonics of load current. IEEE Trans. Magn..

[7] Moses P.S., Masoum M.A.S. (2012). Three-phase asymmetric transformer aging considering voltage-current harmonic interactions, unbalanced nonlinear loading, magnetic couplings, and hysteresis. IEEE Trans. Energy Convers..

[8] Maeda H., Harada K., Ishihara Y. (1996). Performance of the magnetostriction of a silicon steel sheet with a bias field. J. Magn. Magn. Mater..

[9] Jang P., Choi G. (2012). Acoustic noise characteristics and magnetostriction of fe-si powder cores. IEEE Trans. Magn..

[10] Ben T., Yang Q.X., Yan R.G., Zhu L.H. (2017). Magnetically controlled saturable reactor core vibration under practical working conditions. IEEE Trans. Magn..

[11] Ebrahimi H., Gao Y., Kameari A. (2013). Coupled magneto-mechanical analysis considering permeability variation by stress due to both magnetostriction and electromagnetism. IEEE Trans. Magn..

[12] Gao Y., Muramatsu K., Hatim M.J. (2011). The effect of laminated structure on coupled magnetic field and mechanical analyses of iron core and its homogenization technique. IEEE Trans. Magn..

[13] Yamagashira M., Wakabayashi D., Enokizono M. (2014). Vector magnetic properties and 2-d magnetostriction of various electrical steel sheets under rotating flux condition. IEEE Trans. Magn..

[14] Moses A.J. (2003). Measurement of magnetostriction and vibration with regard to transformer noise. IEEE Trans. Magn..

[15] Bai B.D., Wang J.Y. (2014). Research on magnetostriction of grain-oriented electrical silicon-steel sheet. Compel-Int. J. Comp. Math. Electr. Electron. Eng..

[16] Mogi H., Matsuo Y., Kumano T. (1999). Ac magnetostriction hysteresis and magnetization direction in grain oriented silicon steel. IEEE Trans. Magn..

[17] Tanzer T., Pregartner H., Labinsky R. (2018). Magnetostriction of electrical steel and its relation to the no-load noise of power transformers. IEEE Trans. Ind. Appl..

[18] Chang Y.H., Hsu C.H., Chu H.L. (2011). Magnetomechanical vibrations of three-phase three-leg transformer with different amorphous-cored structures. IEEE Trans. Magn..

[19] Rivera H.L., Garcia-Souto J.A., Sanz J. (2000). Measurements of mechanical vibrations at magnetic cores of power transformers with fiber-optic interferometric intrinsic sensor. IEEE J. Sel. Top. Quantum Electron..

[20] Moses A.J., Anderson P.I., Somkun S. (2015). Modeling 2-D Magnetostriction in nonoriented electrical steels using a simple magnetic domain model. IEEE Trans. Magn..

[21] Somkun S., Moses A.J. Quantification of Magnetostriction for Analysis of Vibration of Electrical Machine Cores. Proceedings of the 45th International Universities Power Engineering Conference (UPEC2010).

[22] Somkun S., Moses A.J. (2010). Magnetostriction anisotropy and rotational magnetostriction of a nonoriented electrical steel. IEEE Trans. Magn..

[23] Vandevelde L., Hilgert T.G.D., Melkebeek J.A.A. (2004). Magnetostriction and magnetic forces in electrical steel: Finite element computations and measurements. IEEE Proc. Sci. Meas. Technol..

[24] Zhu L., Yoon H.S. (2016). Finite-element analysis of magnetostriction force in power transformer based on the measurement of anisotropic magnetostriction of highly grain-oriented electrical steel sheet. IEEE Trans. Magn..

[25] Kai Y., Tsuchida Y., Todaka T. (2012). Measurement of the two-dimensional magnetostriction and the vector magnetic property for a non-oriented electrical steel sheet under stress. J. Appl. Phys..

[26] Vandevelde L., Gyselinck J., Wulf M.A.C.D. (2004). Finite-element computation of the deformation of ferromagnetic material taking into account magnetic forces and magnetostriction. IEEE Trans. Magn..

[27] Hilgert T., Vandevelde L., Melkebeek J. (2008). Comparison of magnetostriction models for use in calculations of vibrations in magnetic cores. IEEE Trans. Magn..

[28] Zhu L., Yang Q., Yan R. (2015). Research on dynamic vibration of transformer with wireless power transfer system load. IEEE Trans. Magn..

[29] Hsu C.H., Lee S.L., Lin C.C. (2015). Reduction of vibration and sound-level for a single-phase power transformer with large capacity. IEEE Trans. Magn..

[30] Zhang B., Yan N., Du J.M. (2018). A novel approach to investigate the core vibration in power transformer. IEEE Trans. Magn..

[31] He J.L., Yu Z.Q., Zeng R. (2012). Vibration and audible noise characteristics of AC transformer caused by HVDC system under monopole operation. IEEE Trans. Power Deliv..

[32] Yanase S., Yamamoto K., Okazaki Y. (2001). Measurement of magnetostriction and magnetic properties in an open magnetic circuit. J. Mater. Process. Technol..

[33] Yabumoto M., Arai S., Kawamata R. (1997). Recent development in grain-oriented electrical steel with low magnetostriction. J. Mater. Eng. Perform..

[34] Mogi H., Yabumoto M., Mizokami M. (1996). Harmonic analysis of AC magnetostriction measurements under non-sinusoidal excitation. IEEE Trans. Magn..

[35] Nakata T., Takahashi N., Nakano M. (1994). Magnetostriction measurements with a laser Doppler velocimeter. IEEE Trans. Magn..

[36] Nakase T., Nakano M., Fujiwara K. (2002). Measuring system for magnetostriction of silicon steel sheet under AC excitation using optical methods. IEEE Trans. Magn..

[37] Bán G., Janosi F. (1996). Measuring system and evaluation method of DC and AC magnetostriction behaviour to investigate 3.2% SiFe GO electrical steels. J. Magn. Magn. Mater..

[38] Hirano M., Ishihara Y., Harad K. (2003). A study on measurement of magnetostriction of silicon steel sheet by laser displacement meter. J. Magn. Magn. Mater..

[39] International Electrotechnical Commission (2010). IEC/TR 62581 Electrical Steel-Methods of Measurement of the Magnetostriction Characteristics by Means of Single Sheet and Epstein Test Specimens.

